# Using health check data to investigate cognitive function in Aboriginal and Torres Strait Islanders living with diabetes in the Torres Strait, Australia

**DOI:** 10.1002/edm2.297

**Published:** 2021-09-24

**Authors:** Fintan Thompson, Linton R. Harriss, Sarah Russell, Sean Taylor, Lucette A. Cysique, Edward Strivens, Paul Maruff, Robyn McDermott

**Affiliations:** ^1^ Australian Institute of Tropical Health and Medicine College of Public Health, Medical and Veterinary Sciences James Cook University Cairns Qld Australia; ^2^ College of Medicine and Dentistry James Cook University Cairns Qld Australia; ^3^ Queensland Health, Cairns and Hinterland Hospital and Health Service Cairns Qld Australia; ^4^ Top End Health Service Northern Territory Government Darwin NT Australia; ^5^ School of Psychology University of New South Wales Sydney NSW Australia; ^6^ Cogstate Ltd Melbourne Vic. Australia; ^7^ University of South Australia Adelaide SA Australia

**Keywords:** cognition, complications of diabetes mellitus, indigenous peoples

## Abstract

**Background:**

Type 2 Diabetes (T2DM) has a subtle deleterious effect on cognition and imposes a higher lifetime risk of cognitive impairment and dementia. In populations where both T2DM and dementia are highly prevalent, understanding more about the early effects of T2DM on cognition may provide insights into the lifetime risks of this disease.

**Methods:**

In 2016, 186 Australian Aboriginal and/or Torres Strait Islander residents of the Torres Strait (54% female, mean age =38.9 years, *SD* =15.9, range =15–74) participated in a community health check. The effect of diabetes (Type 1 or Type 2) on speed of thinking and working memory was assessed with the Cogstate Brief Battery (CBB) during the health check.

**Results:**

One third of participants had diabetes (*n* = 56, 30.1%). After adjusting for age, education and previous iPad/Tablet experience, participants with diabetes had a small, yet significant reduction in accuracy on the One Back working memory task (*β* = −.076, *p* = .010, *r* ^2^ = .042). The effect was most pronounced among participants with diabetes aged 20–49 years (*n* = 20), who also had evidence of poorer diabetes control (eg HbA1c% ≥6.5, 76.6%), relative to participants with diabetes aged 50 years and over (*n* = 31) (HbA1c% ≥6.5, 32.0%, *p* = .005).

**Conclusions:**

Early and subtle decrements in working memory may be a potential complication of diabetes among Aboriginal and Torres Strait Islander residents of the Torres Strait. Several potentially influential variables were not captured in this study (eg medication and diabetes duration). Greater preventative health resources are required for this population, particularly given the emerging elevated dementia rates linked to chronic disease.

## Graphical abstract

In 2016, 186 Australian Aboriginal and/or Torres Strait Islander residents of the Torres Strait participated in a community health check, which included a cognitive assessment. The results suggest early and subtle decrements in working memory may be a potential complication of diabetes among Aboriginal and Torres Strait Islander residents of the Torres Strait.

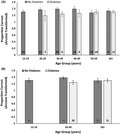

## 1 INTRODUCTION

Type 2 Diabetes (T2DM) is a major global public health concern. In 2017, T2DM was the ninth leading cause of mortality and affected 6.38% of the world's population. This represents a prevalence milestone in a rising trend that shows no signs of abating.^1^ In Australia, T2DM was the 12th largest contributor to the country's 2015 burden of disease and affected 5.3% of adults in 2017–2018.^2^ Among Aboriginal and Torres Strait Islander inhabitants of Australia, referred to here respectfully as Indigenous Australians, the rate of T2DM is 4.3 times higher than in the non‐Indigenous population.^3^

For individuals, T2DM is associated with irreparable damage to multiple body organs and systems, including the brain. For older adults, it is well established that a history of T2DM is associated with a greater likelihood of cognitive impairment and an increased risk for dementia from both cerebrovascular disease and Alzheimer's Disease.^4^ In adults without dementia, cognitive impairment associated with T2DM is subtle and expressed most reliably as decreases in processing speed,^5^ although this may extend to executive function^6^ and working memory.^7^ While studies examining T2DM have been conducted mainly in older adults, qualitatively similar but quantitatively less severe cognitive impairment has been observed in adults with T2DM aged less than 60 years.^8^, ^9^

For Indigenous Australians, the prevalence of dementia is 3–5 times higher than non‐Indigenous Australians.^10^, ^11^, ^12^, ^13^ Elevated levels of childhood trauma, stroke, head injury, and lower levels of skilled employment have been identified as predictors of dementia among mainland Aboriginal populations, while more education has been identified as protective. Recent evidence suggests that vascular risk factors, including diabetes, may be associated with higher rates of dementia among Indigenous Australians living in the Torres Strait.^13^, ^14^ Understanding more about the cognitive implications of diabetes in these vulnerable populations could therefore improve understanding of the development of diabetes‐related brain disease and assist with targeting preventative health activities.

The Zenadth Kes Health Partnership (ZKHP) was a community‐based health‐screening program of residents aged 15–78 years, undertaken on two islands in the Torres Strait between October and December 2016.^15^ In this population, where diabetes incidence is almost four times the general Australian population,^16^ the purpose of the ZKHP was to provide a health service for the community and simultaneously explore the association between the metabolic syndrome and other chronic health conditions. The aim of this current study was to understand whether diabetes, defined as either Type 1 or Type 2, was having a subclinical effect on cognition in this population. It was hypothesized that speed and accuracy on cognitive tasks would reduce as a function of age and increase with years of education and iPad/Tablet experience. It was also hypothesized that participants with diabetes would have slower reaction times and lower accuracy on cognitive tasks, after accounting for age, education and iPad/Tablet experience.

## 2 METHODS

Detailed information about the ZKHP methodology is published elsewhere.^15^, ^17^ A brief overview is provided below. Ethical approval for this study was granted by the Far North Queensland Human Research Ethics Committee (HREC/16/QCH/70–1059).

### 2.1 Participants

Participants were community members aged 15 years and over who identified as being of Torres Strait Islander or Aboriginal descent. A total of 214 participants were screened and met the inclusion criteria (ie consent for both the health check and for the additional research measures). A subset of 14 people met one or more of the exclusion criteria (ie (1) non‐consent, (2) insufficient responses to broader study questions, (3) time constraints or (4) having a physical or sensory disability (eg vision) preventing valid assessment. A total of 200 participants underwent cognitive assessment and the sample for the current study was limited to the 186 participants who had a complete and valid result on at least one of the cognitive tests.^17^ The 14 participants excluded at this stage were older than those who were retained (ie mean age of 62.8 and 38.9 years, respectively, *p *< .001) and more likely to have diabetes (*p *= .002).

### 2.2 Data collection

Demographic information, diet, health behaviours, depressive symptoms and blood samples for several routine and research blood tests were collected. Demographic data were age (years), gender (male, female), total years of education, employment status when of working age (15–64 years) defined as a person having a paid job (yes, no) and island of residence. Information on history of use of electronic devices (iPad or computer tablet (yes, no)) and hand dominance was also collected. Self‐reported use of alcohol and tobacco was collected, and participants also completed a food questionnaire, which included consumption of take‐away food and sugary drinks in the week preceding their health check.

Measures of cardiovascular health included heart rate, systolic and diastolic blood pressure, hypertension (ie systolic ≥140 mmHg or diastolic ≥90 mmHg) and urinary albumin creatinine ratio (urinary ACR). Metabolic markers were triglycerides, high‐density lipoprotein (HDL), low‐density lipoprotein (LDL), total cholesterol, HDL/total cholesterol ratio, glucose, glycosylated haemoglobin (HbA1c%), body mass index (BMI), waist circumference (centimetres), waist/height ratio. Glucose, triglycerides, lipoproteins and cholesterol were analysed at a commercial pathology service (Sullivan Nicolaides, Cairns, Australia). A measure of omega‐3 (*n *− 3) long‐chain polyunsaturated fatty acid (LCPUFAs) was obtained by a whole blood collection on a validated dried blood spot system. Fatty acid composition was analysed by capillary gas chromatography.^18^

An adapted Patient Health Questionnaire 9 (aPHQ‐9) depression screening tool was used to measure depressive symptoms. This instrument has been specifically designed for use with Indigenous Australians in primary healthcare settings.^19^

### 2.3 Diabetes

Participants were defined as having diabetes if they self‐reported being treated for diabetes or had a HbA1c greater than or equal to 6.5% based on blood pathology analyses. The definition of diabetes used in this study encompassed both Type 1 and Type 2. The presence of gestational diabetes (GDM) was not captured. However, given that T2DM is highly prevalent in this population,^16^ it likely accounted for almost all of the diabetes cases. Participants who did not report being treated for diabetes and had a HbA1c ≥ 6.5% were considered as ‘newly diagnosed diabetes’. Information on which medication participants with diabetes had been prescribed was obtained by reviewing participant medical records. Collection of this data was limited to participants from only one of the island sites. As a result, medication information was available for approximately half of the participants with diabetes and was not included in the analyses.

### 2.4 Cognitive assessment

Cognition was assessed in the study using the Cogstate Brief Battery (CBB), using an iPad platform, predominantly by a member of the team who was a provisional psychologist (FT). The CBB was selected as the cognitive screening tool as it has high sensitivity to cognitive dysfunction associated with dementia and MCI.^20^ The tool also has some usability, acceptability and validity among Indigenous Australians,^21^, ^22^, ^23^ including Indigenous residents of the Torres Strait.^17^ Further details on the appropriateness of the CBB and its administration during the current study are described elsewhere.^17^

A description of the four CBB tasks and their ‘built‐in’ Completion and Integrity Criteria is provided in Table S1. In brief, the four tasks comprise; (1) Detection, (2) Identification, which are both measures of attention and processing speed, (3) One Card Learning, a measure of visual memory and (4) One Back Learning, a visual measure of working memory. A CBB task was considered ‘complete and valid’ if the task was completed and the results were valid based on examiner observations and Cogstate Integrity Criteria.^17^

### 2.5 Outcome measures

The primary outcome measures in this study were reaction time and accuracy on the CBB tasks. The Detection, Identification and One Back tasks provided three measures of reaction time in milliseconds and log_10_ transformed milliseconds. The One Card Learning and One Back tasks provided two measures of accuracy as a percentage and an Arcsine transformed percentage. In addition, each participant had a z‐score derived for each of these transformed measures, using normative data in provided by Cogstate PTY LTD. The normative data comprised means and standard deviations for the four CBB tasks for males and females combined by six age groups (18–34, 35–49, 50–59, 60–69, 70–79 and 80–89). Z‐scores that were one standard deviation (SD) below the respective Cogstate mean were flagged using binary yes/no variable (ie z‐score < 1SD), as were z‐score that were two SDs below the mean (ie z‐score < 2SD). In addition, scores that were 0.5 SD below the mean were also flagged, as this cut‐off has been used elsewhere as a more sensitive measure of cognitive impairment.^7^

For each participant, a ‘cognitive speed’ domain was derived (ie an average log‐transformed reaction time measure from three tasks with reaction time measures). Similarly, an average Arcsine transformed accuracy measure was derived for each participant from the two tasks with accuracy measures. Only participants with complete and valid measures on these tasks had the average measures derived.

### 2.6 Statistical analyses

All analyses were undertaken using STATA 15 Statistical software (StataCorp. 2017, College Station, TX). Prior to statistical analysis, the distribution of the data was assessed for normality assumptions and outliers. The distribution of study variables (eg demographic and cardio‐metabolic) by diabetes status was tested with independent‐sample t tests and rank‐sum tests for parametric and non‐parametric continuous data, respectively, and with Chi2 tests for categorical data (Table S2). For the CBB outcome variables, the distribution of parametric measures (ie log‐transformed reaction times and Arcsine transformed accuracy) was examined by diabetes status using means and linear regressions (Table S3). Nonparametric CBB outcomes (ie untransformed reaction time and accuracy measures, number of errors and continuous z‐scores) were examined by diabetes status using medians and Wilcoxon rank‐sum tests. Binary variables that identified whether a Z‐score was one or two standard deviations below a Cogstate mean were examined by diabetes status using proportions and Pearson chi‐squared tests.

**FIGURE 1:**
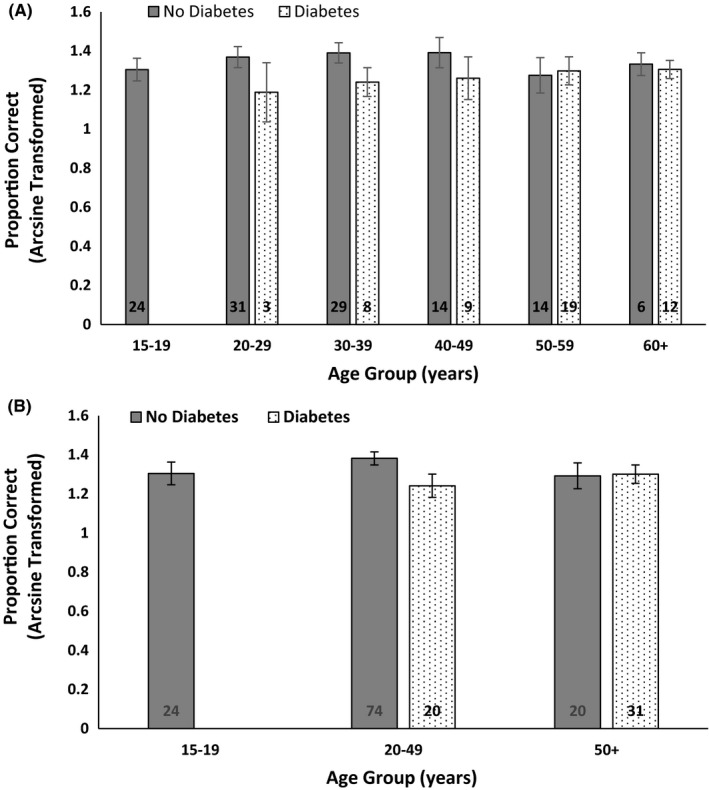
Arcsine transformed proportion of correct responses to the Cogstate Brief Battery One Back working memory test with 95% confidence intervals, by 10‐year age group (Figure 1A) and three age groups (Figure 1B) by diabetes status with number of participants displayed, among Torres Strait Islanders attending the 2016 Zenadth Kes Health Partnership health screen.

Univariate regression analyses were used to examine the distribution of CBB outcome variables by study variables (eg demographic, cardio‐metabolic and immune markers; Table 1). As age, education and iPad/Tablet experience were all associated with CBB outcomes and diabetes status at a univariate level, these factors were adjusted for in three multivariate regression models examining the relationship between diabetes and each CBB outcome (Table 2). Specifically, Model 1—unadjusted, Model 2—adjusted for age, Model 3—adjusted for age and education, Model 4—adjusted for age, education and previous iPad/Tablet use. Effect size for the regression models was calculated using STATA’s ‘estat esize’ post hoc command. The distribution in One Back Arcsine transformed accuracy measures with confidence intervals, by different age groups (ie 10‐year age groups and three broader age groups) and diabetes status, is provided in Figure 1 and Figure S1. Interaction terms were used to analyse the effect of diabetes on One Back outcomes, within each of these age groups, unadjusted and adjusted for education and previous iPad/Tablet use. *p* Values < 0.05 were considered statistically significant.

**TABLE 1:** Performance on selected Cogstate Brief Battery tasks by selected study variables, among 186 Torres Strait Islanders who attended the 2016 Zenadth Kes Health Partnership health screen and completed a Cogstate Brief Battery task.

Variable	Values	Detection Speed					Identification Speed					One Back Accuracy				
		*N*	Mean	(95%CI)	*b*	*p*	*N*	Mean	(95% CI)	*b*	*p*	*N*	Mean	(95% CI)	*b*	*p*
Demographic Information		183					181					169				
Age	(years)				0.002	0.000				0.002	0.000				−0.001	0.178
Gender	Male	84	2.54	(2.52,2.56)			79	2.71	(2.69,2.72)			73	1.32	(1.29,1.36)		
	Female	99	2.52	(2.51,2.54)	−0.013	0.341	102	2.69	(2.68,2.70)	−0.019	0.051	96	1.33	(1.30,1.36)	0.008	0.729
Highest education	Some primary	8	2.63	(2.46,2.80)			9	2.77	(2.68,2.86)			6	1.31	(1.23,1.40)		
	Some secondary	56	2.54	(2.52,2.57)	−0.086	0.014	54	2.70	(2.69,2.72)	−0.063	0.004	52	1.32	(1.28,1.36)	0.003	0.964
	Secondary	60	2.51	(2.49,2.53)	−0.119	0.001	59	2.68	(2.66,2.69)	−0.089	0.000	54	1.32	(1.28,1.36)	0.007	0.914
	Tertiary	57	2.53	(2.50,2.55)	−0.102	0.004	58	2.69	(2.68,2.71)	−0.073	0.001	56	1.35	(1.31,1.39)	0.039	0.544
Anthropometry																
Body Mass Index	(kg/m^2^)				0.000	0.621				0.001	0.182				−0.002	0.202
Waist circumference	(cm)				0.001	0.189				0.001	0.001				−0.001	0.062
Waist/Height ratio					0.084	0.182				0.130	0.002				−0.207	0.046
Cardio‐metabolic profile																
HbA1c (%)	≥6.5	23	2.58	(2.52,2.64)			23	2.70	(2.68,2.73)			21	1.29	(1.22,1.37)		
	<6.5	135	2.53	(2.51,2.54)	−0.052	0.016	132	2.69	(2.68,2.70)	−0.012	0.415	124	1.33	(1.30,1.36)	0.038	0.295
Diabetes	No	129	2.52	(2.50,2.53)			125	2.69	(2.68,2.70)			118	1.35	(1.32,1.38)		
	Yes	54	2.56	(2.53,2.59)	0.041	0.007	56	2.72	(2.70,2.73)	0.030	0.004	51	1.28	(1.24,1.32)	−0.073	0.003
Hypertension	No	143	2.52	(2.51,2.54)			139	2.69	(2.68,2.70)			133	1.34	(1.31,1.36)		
	Yes	39	2.55	(2.52,2.59)	0.030	0.077	41	2.71	(2.69,2.74)	0.024	0.032	35	1.31	(1.25,1.36)	−0.030	0.298
Cholesterol/HDL ratio^a^	0.0–4.5	106	2.53	(2.51,2.55)			102	2.69	(2.68,2.70)			99	1.34	(1.31,1.37)		
	>4.5	60	2.54	(2.51,2.56)	0.009	0.579	61	2.70	(2.69,2.72)	0.013	0.190	54	1.29	(1.25,1.33)	−0.050	0.048
Other factors																
Used iPad/Tablet	No	46	2.57	(2.53,2.60)			46	2.72	(2.70,2.74)			38	1.30	(1.25,1.36)		
	Yes	136	2.52	(2.50,2.53)	−0.048	0.003	135	2.69	(2.68,2.70)	−0.027	0.013	131	1.34	(1.31,1.36)	0.031	0.259
Omega 3 Index					0.009	0.128				0.012	0.001				−0.003	0.753

^a^ Ratio of Total Cholesterol and High‐Density Lipoprotein (HDL) cholesterol.

**TABLE 2:** Multivariate Regression models of selected Cogstate Brief Battery tasks by selected study variables, among 186 Torres Strait Islanders who attended the 2016 Zenadth Kes Health Partnership health screen and completed a Cogstate Brief Battery task.

Variable	Values	Detection Speed				Identification Speed				One Back Accuracy			
		Model 1		Model 4		Model 1		Model 4		Model 1		Model 4	
		b^a^	p^a^	b^a^	p^a^	b^a^	p^a^	b^a^	p^a^	b^a^	p^a^	b^a^	p^a^
Diabetes	No												
	Yes	0.041	0.007	0.013	0.436	0.030	0.004	0.002	0.836	−0.073	0.003	−0.076	0.010
Demographic Information													
Age	(years)	0.002	0.000	0.002	0.002	0.002	0.000	0.002	0.000	−0.001	0.178	0.000	0.889
Education	Some secondary (reference)												
	Secondary	−0.044	0.009	−0.044	0.005	−0.035	0.002	−0.033	0.002	0.004	0.879	0.009	0.762
	Tertiary	−0.027	0.116	−0.039	0.017	−0.019	0.105	−0.029	0.007	0.037	0.194	0.050	0.078
iPad/Tablet	No (ref)												
	Yes	−0.048	0.003			−0.027	0.013	−0.003	0.762	0.031	0.259	0.008	0.791
Other measures													
Body Mass Index	(kg/m^2^)	0.000	0.621	−0.001	0.216	0.001	0.182	0.001	0.386	−0.002	0.202	−0.001	0.776
Waist/Height ratio		0.084	0.182	−0.027	0.683	0.130	0.002	0.063	0.131	−0.207	0.046	−0.084	0.483
HbA1c (%)		0.013	0.007	0.006	0.285	0.007	0.030	0.003	0.364	−0.005	0.531	0.008	0.451
Hypertension	No (reference)												
	Yes	0.030	0.077	0.006	0.724	0.024	0.032	0.006	0.560	−0.030	0.298	−0.019	0.527
Total Chol/HDL ratio^b^		−0.002	0.791	0.000	0.976	0.003	0.474	0.003	0.342	−0.017	0.080	−0.019	0.055
Omega 3	Index	0.009	0.128	−0.006	0.306	0.012	0.001	0.003	0.489	−0.003	0.753	0.014	0.196
Handedness	Left (reference)												
	Right	−0.071	0.003	−0.068	0.003	−0.015	0.370	−0.017	0.262	−0.022	0.599	−0.037	0.393
aPHQ Referred^c^	No (reference)												
	Yes	0.007	0.755	0.035	0.126	−0.010	0.525	0.010	0.463	0.023	0.555	0.007	0.871

Model 1—Unadjusted univariate regression, Model 4—Adjusted for age, education and previous iPad/Tablet experience. Models 2 and 3 are not displayed in this table.

^a^ Beta coefficient.

^b^ *p*‐value.

^c^ Ratio of Total Cholesterol and High‐Density, Lipoprotein (HDL) cholesterol.

^d^ Adapted Patient Health Questionnaire 9.

## 3 RESULTS

### 3.1 Participants

There were 186 participants (54% female, mean age = 38.9 years, SD = 15.9, range = 15–74). Approximately one third (*n* = 56, 30.1%) had diabetes (Table S1), either as an existing condition (*n* = 49) or newly diagnosed (*n* = 7) at assessment. Compared to people without diabetes, those with diabetes were approximately 17 years older (*p *< .001), more likely to have tertiary education (40.0% and 28.7%, respectively, *p *= .015), had higher BMI (*M* = 34.6 and 30.6, respectively, *p *< .001), greater waist/height ratio (*M* = 0.70 and 0.61, respectively, *p *< .001), higher urinary ACR (Table S2), and less likely to have previous iPad/Tablet experience (60.7%) than those without (79.8%, *p *= .006). The number of CBB tasks completed was equivalent between the groups.

### 3.2 Univariate analyses

When performance on CBB tasks was examined in univariate analyses, participants with diabetes were significantly slower on all reaction time measures and had significantly lower accuracy on the One Back task (Table 1). Reaction times on the three measures increased as a function of age and decreased with years of education. Previous experience with an iPad/Tablet was associated with faster response times. Accuracy on One Card Learning task was not related to any study variables and these results are not tabled.

### 3.3 Multivariate analyses

After adjusting for age, education and previous iPad/Tablet experience (Model 4), none of the three reaction time measures were significantly associated with diabetes status (Table 2) and neither was the derived ‘cognitive speed’ domain (data not tabled). Participants with diabetes had significantly lower accuracy on the One Back task after adjusting for age, education and previous iPad/Tablet experience (Model 4). Post hoc effect size estimates showed diabetes accounted for approximately 4% of the variance in One Back accuracy in this model. As shown in Table S3, the median number of errors on the One Back task was higher in the diabetes group compared to the no diabetes group (3 and 2, respectively, *p *= .008) and the median z‐score for accuracy on the One Back task was lower among participants with diabetes (−0.39 and 0.09, respectively, *p *= .002). The proportion of participants with a z‐score ≤ 0.05 standard deviations below the age normative mean was also higher among people with diabetes (47.1% and 30.2%, respectively, *p *= .039; Table S3). There were no significant differences in z‐score measures on the other tasks. Other cardiovascular risk factors, HbA1c, health behaviours, depression and gender were not related to Cogstate outcomes during univariate and/or multivariate modelling (Table 2).

Figure 1 shows the Arcsine transformed proportion of correct responses by diabetes status and age group on the One Back working memory task. Younger participants with diabetes tended to perform worse than participants without diabetes in all age groups up to 50 years. From 50 years of age onward, the accuracy was comparable irrespective of diabetes status. The same trend was evident when average One Back Accuracy z‐scores were graphed by age groups (Figure S1). Multiple regression analyses of One Back accuracy with interaction terms for diabetes and the different age groupings were used to further examine the trends in Figure 1. After adjustment for education and iPad/Tablet experience, there were significant associations in the 20–29, 30–39 and 40–49 age groups, albeit with wide confidence intervals and small cell sizes, compared to the reference group 50 years and over (data not tabled). A post hoc power calculation using G*Power software indicated these analyses were underpowered (ie 1‐*β* = 68.7%). Using three age groups instead, there was a significant association in the 20–49 year age group (*β* = −.16, 95% CI: −0.27, −0.05, *p *= .005) and a post hoc power calculation was adequate (ie >80%). This suggests the effect of diabetes on One Back accuracy in this younger age group was significantly different compared to the reference group 50 years and over (data not tabled). Other interaction analyses were not significant.

Table 3 shows younger participants with diabetes (ie 20–49 years, *n* = 21) had higher levels of morbidity compared to older participants with diabetes (ie 50+ years, *n* = 31). Specifically, younger participants with diabetes were more likely to be current smokers (65% and 9.7%, respectively, *p *< .001) and to have consumed a sugary drink in the preceding week (80.0% and 38.7%, respectively, *p *= .004). They were also more likely to be newly diagnosed with diabetes (25.0% and 6.4%, respectively), have a HbA1c greater than 6.5% (76.5% and 32.0%, respectively, *p *= .005) and have a higher Total/HDL ratio (4.82 and 3.85, respectively *p *= .011).

**TABLE 3:** Comparison of Zenadth Kes Health Partnership participants with diabetes who completed the One Back Cogstate Brief Battery task, by age group, 2016 (*n* = 51).

Variable	Values	Diabetes (20–49 years old)			Diabetes (50+years)			*p*
		No.	(%),mean	(95% CI)	No.	(%),mean	(95% CI)	
Demographics	Total	20			31			
Gender	Female	13	(65.0)	(43.6, 86.4)	20	(64.5)	(47.3, 81.8)	.972
Education	At least some primary			(0.0, 0.0)	4	(12.9)	(0.8, 25.0)	.374
	Some secondary	4	(21.1)	(2.3, 39.8)	5	(16.1)	(2.9, 29.4)	
	Completed secondary	7	(36.8)	(14.6, 59.1)	8	(25.8)	(10.0, 41.6)	
	Tertiary	8	(42.1)	(19.3, 64.9)	14	(45.2)	(27.2, 63.1)	
Risk Behaviours	Consumes alcohol	15	(75.0)	(55.6, 94.4)	16	(51.6)	(33.6, 69.6)	.095
Smoker	Never	2	(10.0)	(−3.5, 23.5)	10	(32.3)	(15.4, 49.1)	.000
	Former	5	(25.0)	(5.6, 44.4)	18	(58.1)	(40.3, 75.9)	
	Current	13	(65.0)	(43.6, 86.4)	3	(9.7)	(−1.0, 20.3)	
	Takeaway (prev. week)	7	(35.0)	(13.6, 56.4)	9	(29.0)	(12.7, 45.4)	.654
	Sugary drink yesterday	16	(80.0)	(62.0, 98.0)	12	(38.7)	(21.1, 56.3)	.004
Anthropometry	Body Mass Index (h/kg^2^)	20	34.78	(31.9, 37.6)	31	35.45	(33.5, 37.4)	.682
	Waist/Height ratio	20	0.69	(0.6, 0.7)	30	0.71	(0.7, 0.7)	.221
Cardio‐metabolic	HbA1c (%) ≥6.5	13	(76.5)	(55.7, 97.2)	8	(32.0)	(13.2, 50.8)	.005
	Hypertension	5	(26.3)	(6.0, 46.6)	10	(32.3)	(15.4, 49.1)	.656
	Total Cholesterol	18	4.93	(4.4, 5.5)	27	4.38	(4.0, 4.8)	.087
	HDL Cholesterol^d^	18	1.09	(0.9, 1.2)	27	1.21	(1.0, 1.4)	.144
	LDL Cholesterol^a^	17	2.64	(2.2, 3.1)	27	2.33	(2.0, 2.7)	.255
	Total/HDL Ratio^b^	18	4.82	(4.0, 5.6)	27	3.85	(3.4, 4.3)	.011
	Omega 3 Index	20	5.94	(5.4, 6.4)	31	7.06	(6.6, 7.6)	.001
Other factors	aPHQ (referred)^c^	2	(10.0)	(−3.5, 23.5)	1	(3.2)	(−3.1, 9.6)	.315
	Used iPad/Tablet	14	(70.0)	(49.4, 90.6)	18	(58.1)	(40.3, 75.9)	.389
	ONB Accuracy ‐ Trans.	20	(1.2)	(1.2, 1.3)	31	(1.3)	(1.3, 1.4)	.105

Other		No.	Median	(IQR)	No.	Median	(IQR)	*p*
	ONB Accuracy ‐ Untrans.	20	0.91	(0.81, 0.94)	31	0.94	(0.86, 0.97)	.116
	ONB Errors (No.)	20	3.00	(2.00, 7.50)	31	2.00	(1.00, 5.00)	.148

^a^ High‐Density Lipoprotein (HDL).

^b^ Low‐Density Lipoprotein (LDL) cholesterol.

^c^ Ratio of Total Cholesterol and HDL cholesterol.

^d^ Adapted Patient Health Questionnaire 9.

## 4 DISCUSSION

This study examined relationships between diabetes and cognition, measured by the speed and accuracy of performance on a standardized test battery, in Indigenous Australians living in the Torres Strait. After accounting for age, education and previous iPad/Tablet experience, participants with diabetes had significantly lower accuracy on a task of working memory compared to those without, although the absolute difference between the groups and effect size was both very small. Younger participants (ie 20–49 years of age) with diabetes had lower accuracy scores on this task and evidence of greater morbidity compared to participants 50 years and older with diabetes. As hypothesized, age, education and iPad/Tablet experience were associated with reaction time measures for all participants. There were no differences in speed measures between the participants with and without diabetes after adjusting for these variables.

The One Back task used in the current study is a measure of working memory, which is a cognitive ability underpinned by executive functioning.^24^ Our results therefore correspond with research showing lower working memory^7^ and executive function^6^, ^25^ among people with T2DM. As the reduction we observed was primarily among participants in their young to mid‐adult life (ie 20–49 years), our results are most consistent with findings from a recent meta‐analysis of 12 studies that showed working memory and executive functioning decrements are observable in midlife among people with T2DM.^9^ Our study supports a growing consensus that cognition may be affected in early to mid‐adulthood during T2DM and adds to existing knowledge by showing this may represent a potential complication of diabetes for Indigenous residents of the Torres Strait.

The magnitude of reduction observed in One Back accuracy was much lower than reported elsewhere. For example, while Pelimanni and Jehkonen^9^ reported medium effect sizes, in our study, the effect was small. Diabetes status accounted for approximately 4% of the variance in One Back performance and participants with diabetes made, on average, one additional error on this task compared to participants without diabetes. This very modest reduction in performance would be considered, at most, a “diabetes associated cognitive decrement”.^4^ These are subtle decrements that may give rise to self‐reported complaints, but are unlikely to affect social or occupational functioning or diabetes self‐management.^26^ These decrements have been noted to progress slowly over many years,^27^, ^28^ particularly in the context of chronically elevated blood glucose.^6^, ^29^ While this may eventually lead to deficits that are clinically or practically important for everyday functioning,^30^ it remains unclear whether dementia represents an ‘end point’ on the same continuum.^4^ Among older Indigenous residents of the Torres Strait, vascular risk factors such as diabetes have been identified as potential drivers of the elevated dementia rates.^14^ The results of the current study may provide some evidence of the detrimental effect of a vascular risk early in this process. However, this proposition remains far from conclusive given the small effect size in our study and the broader uncertainty about the continuum between diabetes‐associated cognitive decrements and later dementia.

The difference in One Back accuracy between participants with and without diabetes was most evident in younger age groups and became less pronounced with older age. There may be several study design reasons for this finding. First, Indigenous Australians have an earlier age of mortality, which has resulted in a healthy survivor effect in other research.^31^, ^32^ In our study, older participants with diabetes were relatively healthy compared to their younger diabetes counterparts in terms of cardio‐metabolic indicators. Our results suggest a healthy survivor effect could extend to cognition, at least in terms of working memory. Our results may have also been influenced by selection bias, where older residents with diabetes and cognitive difficulties may have been less likely to self‐select into a community health screen initially. We also excluded data from 14 participants who were unable to complete any of the CBB tasks, most of whom were older and had diabetes. Had these participants been retained and appropriately supported to undertake the CBB tasks, as noted elsewhere,^17^ then poorer performance on the One Back task among people with diabetes may have also been observed in the older age groups.

In addition to study design factors, the lower performance of younger participants with diabetes on the One Back task may reflect their increased morbidity relative to their older counterparts. Poorer glycaemic control in the younger group, as evidenced by higher HbA1c, was particularly evident. A systematic review of 86 articles indicated that high HbA1c had a weak negative association with cognitive function in older people (ie 51–85 years of age) with T2DM without dementia.^33^ Our study adds to the body of knowledge by suggesting this effect may also be present among younger people who have both diabetes and notable indicators of morbidity. Our limited sample size of 51 people with diabetes, unfortunately, prevented us from exploring this with further modelling.

In contrast to many studies,^9^ we found no difference in processing speed, as measured by the Detection and Identification tasks, by diabetes status. The requirements of the CBB tasks may be a reason for these contrary results. While these tasks have criterion validity with traditional measures of processing speed, such as the Symbol Digit Modality Test (SDMT) and Grooved Pegboard,^34^ there are notable differences. For example, the CBB speed measures lack an ‘executive function’ component, which is present in the SDMT, in the form of working memory and associative learning memory.^35^ The CBB measures also have a relatively minor ‘motor’ component, which is a notable domain measured by the Grooved Pegboard test.^25^ Further research would be required to examine this possibility. In the absence of this evidence, the current results suggest the CBB tasks are unlikely to be appropriate to detect the early processing speed decrements often seen in diabetes.

In terms of strengths, the examiner was blinded to the diabetes status of participants, which reduced the chance of assessment bias. We obtained diabetes status from both self‐report and HbA1c measures. The results of this study suggest that targeted education for younger people with diabetes and improved diabetes monitoring and management may be valuable in terms of protecting later cognitive health, particularly in contexts where there are higher rates of dementia linked to chronic disease. To our knowledge, this is the first study to indicate a detrimental effect of diabetes using a brief computer‐based cognitive screen. Further research in other communities would be required to verify these early findings.

There were several study limitations. As noted, the high likelihood of selection bias would mean the sample might not be representative of diabetes and cognition in older age groups. Results from other studies suggest diabetes control, evidenced by HbA1c levels, is associated with cognition in older age groups.^33^ Due to small numbers in the older age group, we could not examine the effect of diabetes control in these older ages. Our study also had limited information about medication prescribing and no information about adherence. Year of diagnosis with diabetes was only available in a handful of cases. Duration of diabetes is important, as time with the disease increases risk of cognitive impairment.^6^ The small sample size reduced our confidence in regression modelling with interaction terms for multiple age groups. While aggregating participants into three age groups improved this modelling, it prevented examining differences between the younger age groups. This study also did not differentiate by diabetes into Type 1 and 2 Diabetes and GDM. However, given that T2DM is particularly prevalent in this population,^16^ it is likely this accounted for most of the diabetes cases.

## 5 CONCLUSIONS

In a global context where diabetes remains an important risk factor for cognitive decline, our findings suggest that early and subtle decrements in working memory may be a potential complication of diabetes among Indigenous Australians living in the Torres Strait. In this population, which has elevated dementia rates linked to chronic disease, our results highlight the need for more preventative health resourcing. Our results suggest that early identification of younger people with diabetes, targeted education and supported glycaemic control could be important for protecting cognitive health.

## CONFLICT OF INTEREST

P.M. is a full‐time employee of CogState Ltd. CogState Ltd. provided the CogState tasks reported in this study. The remaining authors declare no conflicts of interest.

## AUTHOR CONTRIBUTIONS

**Fintan Thompson:** Data curation (Lead); Formal analysis (Lead); Investigation (Supporting); Methodology (Equal); Writing‐original draft (lead); Writing‐review & editing (lead). **Linton R. Harriss:** Investigation (equal); Methodology (equal); Project administration (equal); Writing‐original draft (supporting); Writing‐review & editing (supporting). **Sarah Russell:** Formal analysis (supporting); Writing‐original draft (supporting); Writing‐review & editing (supporting). **Sean Taylor:** Conceptualization (equal); Investigation (equal); Methodology (equal); Project administration (equal); Writing‐original draft (supporting). **Lucette A. Cysique:** Formal analysis (supporting); Methodology (supporting); Resources (supporting); Software (supporting); Writing‐original draft (supporting). **Edward Strivens:** Writing‐original draft (supporting); Writing‐review & editing (supporting). **Paul Maruff:** Methodology (supporting); Resources (supporting); Software (supporting); Writing‐original draft (supporting); Writing‐review & editing (supporting). **Robyn McDermott:** Conceptualization (lead); Funding acquisition (lead); Investigation (lead); Methodology (lead); Project administration (lead); Resources (supporting); Writing‐original draft (supporting); Writing‐review & editing (supporting).

## Supporting information

Fig S1

Table S1‐S3

## Data Availability

The data that support the findings of this study are not publicly available due to privacy or ethical restrictions. The data are available on request from the corresponding author. Additional institutional approvals, such as ethics approval, would be required to enable sharing of these data.

## References

[1] Khan MAB , Hashim MJ , King JK , Govender RD , Mustafa H , Al KJ . Epidemiology of type 2 diabetes–global burden of disease and forecasted trends. J Epidemiol Glob Health. 2020;10(1):107‐116. 32175717 10.2991/jegh.k.191028.001 PMC7310804

[2] AIHW . (2020). Australian Institute of Health and Welfare. Diabetes. Cat. no. CVD 82. In: Welfare AIoHa (ed.) AIHW. Viewed 08 December 2020. Retrieved from https://www.aihw.gov.au/reports/diabetes/diabetes

[3] AIHW (2020). Australian Institute of Health and Welfare. Diabetes. 15 Jul 2020. Retrieved from https://www.aihw.gov.au/reports/diabetes/diabetes/contents/hospital‐care‐for‐diabetes/type‐2‐diabetes#atsi

[4] Biessels GJ , Despa F . Cognitive decline and dementia in diabetes mellitus: mechanisms and clinical implications. Nat Rev Endocrinol. 2018;14(10):591‐604. 30022099 10.1038/s41574-018-0048-7 PMC6397437

[5] Dong Y , Kua ZJ , Khoo EYH , Koo EH , Merchant RA . The utility of brief cognitive tests for patients with type 2 diabetes mellitus: a systematic review. Journal of the Am Med Dir Assoc. 2016;17(10):889‐895. 10.1016/j.jamda.2016.06.010 27461866

[6] West RK , Ravona‐Springer R , Schmeidler J , et al. The association of duration of type 2 diabetes with cognitive performance is modulated by long‐term glycemic control. Am J Geriatr Psychiatry. 2014;22(10):1055‐1059. 24534521 10.1016/j.jagp.2014.01.010 PMC4108577

[7] Wong RHX , Scholey A , Howe PRC . Assessing premorbid cognitive ability in adults with type 2 diabetes mellitus—a review with implications for future intervention studies. Curr Diab Rep. 2014;14(11):547‐554. 25273482 10.1007/s11892-014-0547-4

[8] Yau PL , Javier DC , Ryan CM , et al. Preliminary evidence for brain complications in obese adolescents with type 2 diabetes mellitus. Diabetologia. 2010;53(11):2298‐2306. 20668831 10.1007/s00125-010-1857-y PMC3116653

[9] Pelimanni E , Jehkonen M . Type 2 diabetes and cognitive functions in middle age: A meta‐analysis. J Int Neuropsychol Soc. 2019;25(2):215‐229. 30575498 10.1017/S1355617718001042

[10] Radford K , Mack HA , Draper B , et al. Prevalence of dementia in urban and regional Aboriginal Australians. Alzheimers Dement. 2015;11(3):271‐279. 24985534 10.1016/j.jalz.2014.03.007

[11] Smith K , Flicker L , Lautenschlager NT , et al. High prevalence of dementia and cognitive impairment in Indigenous Australians. Neurology. 2008;71(19):1470‐1473. 18799785 10.1212/01.wnl.0000320508.11013.4f

[12] Li SQ , Guthridge SL , Eswara Aratchige P , et al. Dementia prevalence and incidence among the Indigenous and non‐Indigenous populations of the Northern Territory. Med J Aust. 2014;200(8):465‐469. 24794609 10.5694/mja13.11052

[13] Russell SG , Quigley R , Thompson F , et al. Prevalence of dementia in the Torres Strait. Aust J Ageing. 2021;40(2):1‐8. 10.1111/ajag.12878 33169520

[14] Russell SG , Quigley R , Thompson F , et al. Factors associated with the increased risk of dementia found in the Torres Strait. Australas J Ageing. 2021;1‐9. 10.1111/ajag.12980 34351674

[15] Berger M , Taylor S , Harriss L , et al. Cross‐sectional association of seafood consumption, polyunsaturated fatty acids and depressive symptoms in two Torres Strait communities. Nutr Neurosci. 2020;23(5):353‐362. 30073906 10.1080/1028415X.2018.1504429

[16] McDermott RA , Li M , Campbell SK . Incidence of type 2 diabetes in two Indigenous Australian populations: a 6‐year follow‐up study. Med J Aust. 2010;192(10):562‐565. 20477730 10.5694/j.1326-5377.2010.tb03636.x

[17] Thompson F , Cysique LA , Harriss LR , et al. Acceptability and usability of computerized cognitive assessment among Australian indigenous residents of the torres strait islands. Arch Clin Neuropsychol. 2020;35(8):1288‐1302. 32647858 10.1093/arclin/acaa037

[18] Liu G , Mühlhäusler BS , Gibson RA . A method for long term stabilisation of long chain polyunsaturated fatty acids in dried blood spots and its clinical application. Prostaglandins Leukot Essent Fatty Acids. 2014;91(6):251‐260. 25451557 10.1016/j.plefa.2014.09.009

[19] Group GiRC , Hackett ML , Teixeira‐Pinto A , et al. Getting it Right: validating a culturally specific screening tool for depression (aPHQ‐9) in Aboriginal and Torres Strait Islander Australians. Med J Aust. 2019;211(1):24‐30. 31256439 10.5694/mja2.50212

[20] Maruff P , Lim YY , Darby D , et al. Clinical utility of the cogstate brief battery in identifying cognitive impairment in mild cognitive impairment and Alzheimer’s disease. BMC Psychol. 2013;1(1):1‐11. 25566378 10.1186/2050-7283-1-30 PMC4269990

[21] Dingwall KM , Gray AO , McCarthy AR , Delima JF , Bowden SC . Exploring the reliability and acceptability of cognitive tests for Indigenous Australians: a pilot study. BMC Psychol. 2017;5(1):26. 28768522 10.1186/s40359-017-0195-y PMC5541699

[22] Dingwall KM , Lewis MS , Maruff P , Cairney S . Reliability of repeated cognitive testing in healthy Indigenous Australian adolescents. Aust Psychol. 2009;44(4):224‐234.

[23] Dingwall KM , Lewis MS , Maruff P , Cairney S . Assessing cognition following petrol sniffing for Indigenous Australians. Aust N Z J Psychiatry. 2010;44(7):631‐639. 20560850 10.3109/00048671003627405

[24] Diamond A . Executive functions. Annu Rev Psychol. 2013;64:135‐168. 23020641 10.1146/annurev-psych-113011-143750 PMC4084861

[25] Palta P , Schneider AL , Biessels GJ , Touradji P , Hill‐Briggs F . Magnitude of cognitive dysfunction in adults with type 2 diabetes: a meta‐analysis of six cognitive domains and the most frequently reported neuropsychological tests within domains. J Int Neuropsychol Soc. 2014;20(3):278‐291. 24555960 10.1017/S1355617713001483 PMC4132660

[26] Koekkoek PS , Kappelle LJ , van den Berg E , Rutten GE , Biessels GJ . Cognitive function in patients with diabetes mellitus: guidance for daily care. Lancet Neurol. 2015;14(3):329‐340. 25728442 10.1016/S1474-4422(14)70249-2

[27] Monette MC , Baird A , Jackson DL . A meta‐analysis of cognitive functioning in nondemented adults with type 2 diabetes mellitus. Can J Diabetes. 2014;38(6):401‐408. 24933107 10.1016/j.jcjd.2014.01.014

[28] Yaffe K , Falvey C , Hamilton N , et al. Diabetes, glucose control, and 9‐year cognitive decline among older adults without dementia. Arch Neurol. 2012;69(9):1170‐1175. 22710333 10.1001/archneurol.2012.1117 PMC3752423

[29] van Duinkerken E , Ryan CM . Diabetes mellitus in the young and the old: Effects on cognitive functioning across the life span. Neurobiol Dis. 2020;134:104608. 31494283 10.1016/j.nbd.2019.104608

[30] Norman GR , Sloan JA , Wyrwich KW . Interpretation of changes in health‐related quality of life: the remarkable universality of half a standard deviation. Med Care. 2003;41(5):582‐592. 12719681 10.1097/01.MLR.0000062554.74615.4C

[31] Condon JR , Cunningham J , Barnes T , Armstrong BK . Long‐term trends in cancer mortality for Indigenous Australians in the Northern Territory. Med J Aust. 2004;180(10):504‐507. 15139826 10.5694/j.1326-5377.2004.tb06052.x

[32] Brimblecombe J , Mackerras D , Garnggulkpuy J , et al. Leanness and type 2 diabetes in a population of indigenous Australians. Diabetes Res Clin Pract. 2006;72(1):93‐99. 16260061 10.1016/j.diabres.2005.09.014

[33] Geijselaers SL , Sep SJ , Stehouwer CD , Biessels GJ . Glucose regulation, cognition, and brain MRI in type 2 diabetes: a systematic review. Lancet Diabetes Endocrinol. 2015;3(1):75‐89. 25163604 10.1016/S2213-8587(14)70148-2

[34] Mielke MM , Weigand SD , Wiste HJ , et al. Independent comparison of CogState computerized testing and a standard cognitive battery with neuroimaging. Alzheimers Dement. 2014;10(6):779‐789. 25458308 10.1016/j.jalz.2014.09.001 PMC4273919

[35] Jaeger J . Digit symbol substitution test: the case for sensitivity over specificity in neuropsychological testing. J Clin Psychopharmacol. 2018;38(5):513‐519. 30124583 10.1097/JCP.0000000000000941 PMC6291255

