# Supplementary material for: Using health check data to investigate cognitive function in Aboriginal and Torres Strait Islanders living with diabetes in the Torres Strait, Australia
Source: Endocrinol Diabetes Metab. 2021 Sep 24;5(1):e00297. doi: 10.1002/edm2.297 (PMC8754245; doi:10.1002/edm2.297)
Supplement: Supplementary file 2 — Table S1‐S3 [file EDM2-5-e00297-s001.docx]

**Supplementary Tables**

**Supplementary Table 1 - Description of the four Cogstate Brief Battery tasks used to assess cognition among Torres Strait Islanders attending the 2016 Zenadth Kes Health Partnership health screen.**

| **Cogstate Task** | **Description** | **Primary outcome measure(s)** | **Cogstate completion criteria** | **Cogstate integrity criteria** |
| --- | --- | --- | --- | --- |
| Detection | The participant attends to a card presented at the center of the screen and presses either the *‘Yes’* button with their right hand as fast as they can when the card turns face up and reveals a red joker. This is a reaction time task measuring visual attention and psychomotor function. | Reaction time, measured in log transformed milliseconds. | Participant responded to 75% of trials. | Accuracy of performance greater than 90%. |
| Identification | The participant attends to a card presented at the center of the screen and when the card turns face up, presses *‘Yes’* with their right hand if the card is a red joker or *‘No’* with their left hand if the card is black joker as fast as they can. This is a choice reaction time task measuring visual attention. | Reaction time, measured in log transformed milliseconds. | Participant responded to 75% of trials. | Accuracy of performance greater than 70%. |
| One Card Learning | The participant attends to a deck of playing cards presented at the center of the screen. When the top card turns face up, the participant presses *‘Yes’* with their right hand if they have already seen that card in the deck of cards, or *‘No’* with their left hand if they have not seen that card. Presented cards are ‘reshuffled’ back into the deck. This task measures visual memory and learning. | Accuracy, measured as the arcsine transformed proportion of correct responses of total cards presented. | Participant responded to 75% of trials. | Accuracy of responses greater than chance (i.e., 50%). |
| One Back | The participant attends to a deck of playing cards presented at the center of the screen. When the top card turns face up, the participant presses *‘Yes’* with their right hand if the presented card matches the previous card, and *‘No’* with their left hand if the card does not match. Presented cards are ‘reshuffled’ back into the deck. This task measures working memory using an n-back paradigm. | Reaction time, measured in log transformed milliseconds.  Accuracy, measured as the arcsine transformed proportion of correct responses of total cards presented. | Participant responded to 75% of trials. | Accuracy of performance greater than 70%. |

**Supplementary Table 2 – Demographic, behavioral, cardio-metabolic and inflammatory indicators by diabetes status among 186 Torres Strait Islanders who attended the 2016 Zenadth Kes Health Partnership health screen and completed a Cogstate Brief Battery task**

**Supplementary Table 3 – Cogstate Brief Battery tasks by diabetes status, among Torres Strait Islanders attending the 2016 Zenadth Kes Health Partnership health screen**
